# The Relationship between Ethnic Classroom Composition and Turkish-Origin and German Students' Reading Performance and Sense of Belonging

**DOI:** 10.3389/fpsyg.2016.01071

**Published:** 2016-07-14

**Authors:** Sog Yee Mok, Sarah E. Martiny, Ilka H. Gleibs, Melanie M. Keller, Laura Froehlich

**Affiliations:** ^1^Department of Empirical Educational Research, University of KonstanzKonstanz, Germany; ^2^Department of Psychology, UiT The Arctic University of NorwayTromsø, Norway; ^3^Department of Psychological and Behavioural Science, London School of Economics and Political ScienceLondon, UK; ^4^School of Education, University of SalzburgSalzburg, Austria; ^5^Department of Social Psychology, University of HagenHagen, Germany

**Keywords:** ethnic composition, Turkish migrants, PISA study, reading performance, sense of belonging

## Abstract

Past research on ethnic composition effects on migrant and ethnic majority students' performance has reported inconclusive results: Some studies have found no relationship between the proportion of migrant students in school and students' performance, some revealed positive effects, whereas others showed negative effects of the proportion of migrant students. Most of the studies did not consider whether an increase in the proportion of migrant students in the classroom has different effects on migrant and ethnic majority students' performance. For this reason, the present study (*N* = 9215) extends previous research by investigating the cross-level interaction effect of the proportion of Turkish-origin students in classrooms on Turkish-origin and German students' reading performance with data based on the German National Assessment Study 2008/2009 in the school subject German. In addition, we examined the cross-level interaction effect of Turkish-origin students' proportion on sense of belonging to school for Turkish-origin and German students, as sense of belonging has been shown to be an important predictor of well-being and integration. No cross-level interaction effect on performance emerged. Only a small negative main effect of the Turkish-origin students' proportion on all students' performance was found. As predicted, we showed a cross-level interaction on sense of belonging. Only Turkish-origin students' sense of belonging was positively related to the proportion of Turkish-origin students: The more Turkish-origin students there were in a classroom, the higher Turkish-origin students' sense of belonging. German students' sense of belonging was not related to the ethnic classroom composition. Implications of the results in the educational context are discussed.

## Introduction

In today's societies migration is widespread. In 2012, 1.7 million persons from a non-European country immigrated to a Western European country; the largest number of migrants was reported in Germany (Eurostat, 2014). The integration of migrants into a multicultural society has become one of the biggest challenges of the 21st century (Deaux and Verkuyten, 2014). Integrating migrants is an important societal goal as a full integration facilitates migrants' well-being and participation in society (Phinney et al., 2001). Nevertheless, in many countries ethnic segregation of neighborhoods has increasingly become a problem (Cutler and Glaeser, 1997). One consequence that comes along with ethnically segregated neighborhoods is a high concentration of migrants within certain classrooms and schools. For example, a study based on the 2006 German National PISA Extension Study revealed that approximately one third of the participating 326 classrooms had 30% or more migrant students per classroom (Walter, 2009). Earlier research on the effects of ethnic composition on students' performance has been inconclusive. Whereas some research showed that a higher percentage of migrant students in a school or classroom can have detrimental effects on all students' academic performance (Stanat, 2006), other studies showed that a more diverse composition of students in classrooms, including both ethnic and achievement-related diversity, can have benefits for the performance of migrant students or low-performing students (Lou et al., 1996; Konan et al., 2010). For these reasons, and to add to previous research on ethnic composition effects in the educational context, in the present work we investigate the relationship between the ethnic composition of a specific ethnic group, namely Turkish-origin students (the largest migrant group in Germany; Statistisches Bundesamt, 2012), on classroom level on the performance of Turkish-origin and German students.

However, it is not only important to examine the relationship between ethnic composition and performance, but also its relationship with important psychological variables. One variable which has recently received attention is people's sense of belonging to groups (Osterman, 2000). Research has shown that sense of belonging is positively associated with pro-social behavior (Battistich et al., 2004) and self-esteem (Baumeister and Leary, 1995). Further, migrants' sense of belonging can have a positive impact on well-being during school (Branscombe et al., 1999) which can persist through transitions into new academic environments (Pittman and Richmond, 2007; Iyer et al., 2009), and is positively associated with integration into the host society (Phinney et al., 2001). Taken together, very little is known about whether the relationship between the ethnic composition in classrooms and performance differs for ethnic majority and migrant students in Germany (Stanat, 2006). Moreover, no research has examined the relationship between ethnic composition and sense of belonging for these ethnic groups. The aim of the present research is to close this research gap. Thus, in the present work we investigate the relationship between ethnic classroom composition and performance as well as its relationship with sense of belonging for Turkish-origin and German students in Germany.

## Ethnic classroom composition effects on performance

The first focus of the present research is to investigate the relationship between ethnic classroom composition (i.e., proportion of Turkish-origin students in the classroom) and students' performance. The extent to which the ethnic composition of schools influences students' performance has been investigated in a meta-analysis by van Ewijk and Sleegers (2010) with studies mostly from the USA. The authors investigated both which ethnic groups produced the effects and which groups were affected. Results showed that a high percentage of individuals belonging to a minority ethnic group, for example African Americans, negatively affects the performance of students from the same ethnic group more than students of other ethnic groups. In Europe, studies investigating ethnic composition on migrant and ethnic majority students' performance have shown inconsistent results. On the one hand, some studies have found a positive association with the proportion of migrants on the performance of both ethnic majority and migrant students (Konan et al., 2010). On the other hand, many studies have found either no (Cebolla-Boado and Garrido Medina, 2011; Agirdag et al., 2012) or even negative effects (Stanat, 2006; Verwiebe, 2013) of higher proportions of migrants in the classroom or in the school on all students' performance. However, the majority of past research revealed a negative effect of the proportion of migrants on academic outcomes for specific migrant groups (Westerbeek, 1999; Fekjær and Birkelund, 2007; Contini, 2013). Drawing on the findings of negative composition effects on performance of migrants, we postulate that an increase in the proportion of migrants will be negatively related to migrant students' performance.

### Effects of turkish-origin students' proportion on performance in germany

Among the OECD countries, Germany is one of the countries in which social or ethnic background has the strongest impact on academic achievement (Entorf and Minoiu, 2005) and thus it is an important population in which to investigate specific effects of ethnic composition in the schools. In the present work, we focus on Turkish-origin migrants for several reasons. First, migrants of Turkish origin are the largest group of migrants in Germany (18.5% of the German population; Statistisches Bundesamt, 2012). Second, Turkish-origin students consistently underperform in the German educational system (Klieme et al., 2010) and are much more likely to attend the lowest track school (*Hauptschule;* Baumert and Schümer, 2001). Thus, it seems particularly important to investigate variables that hinder or benefit Turkish-origin students' performance in the German educational system.

Previous research has often not distinguished between the impact of ethnic composition effects on specific ethnic groups. Very few studies have investigated whether composition effects affect the same ethnic group, the ethnic majority group, or all students' performance equally (Stanat, 2006). One exception is a study by Walter and Stanat (2008). These authors showed based on data from the German National PISA Extension Study 2003 that Turkish-origin students' reading performance decreased as the school-level proportion of Turkish-origin students increased. Based on the findings of Walter and Stanat (2008), we expected that ethnic minority students' performance would be negatively related to the proportion of same-ethnic minority students in the classroom.

For ethnic majority students, majority group members' performance has not been shown to suffer from an increased outgroup member concentration (Stanat, 2006). In standardized performance tests ethnic majority students consistently outperformed ethnic minority students (Klieme et al., 2010; Stanat et al., 2010). Thus, ethnic majority students are not likely to be affected by the number of ethnic minority students in classroom. Drawing on these findings, we expected for majority students that their performance is not affected by an increase in the proportion of ethnic minority students in the classroom.

Bringing these findings together, in the present work we concentrate on Turkish-origin students and investigate whether an increase in Turkish-origin students' percentage in the classroom will be differentially related to Turkish-origin and German students' reading performance. In addition, we focus on the composition at the classroom level instead of at the school level as we assume a stronger impact of the daily contact within the classroom on students' performance (Stanat, 2006). Taken together, based on empirical findings revealing negative composition effects for migrants, we expect a cross-level interaction effect between the percentage of Turkish-origin students and students' ethnicity on performance. In detail, we hypothesize an increase in the percentage of Turkish-origin students in the classroom will be negatively related to reading performance for Turkish-origin students, but not for German students.

## Ethnic classroom composition effects on sense of belonging

The second focus of the present work is to investigate the relationship between ethnic classroom composition and students' sense of belonging. The desire for social bonds has a long history in psychological research and has been linked to the need for a positive view from others, which was defined as social belongingness (Baumeister and Leary, 1995). Belongingness is one of the basic human motives, which is needed for well-being (Deci and Ryan, 1991; Baumeister and Leary, 1995). When individuals fail to meet their belonging needs, this failure can lead to long-term disidentification from academic domains (Major et al., 1998). Further, individuals' sense of belonging is positively associated with pro-social behavior (Battistich et al., 2004) and psychological well-being, especially for ethnic minorities (Branscombe et al., 1999). Drawing on these previous findings, we assume that migrant students' sense of belonging is an important proxy for integration and well-being, and we therefore investigate the relationship between classroom composition and Turkish-origin and German students' sense of belonging to school.

### Sense of belonging in the educational context

While sense of belonging has been well-investigated for negatively stereotyped adults in academic domains (Good et al., 2012), little is known about migrant students' sense of belonging to school (but see Cohen et al., 2006; for review, see Osterman, 2000). Goodenow (1993) defined students' sense of belonging to school as the extent to which students feel accepted, included, respected, and valued by others in school. We used this definition in the present study. Studies investigating students' sense of belonging to school have mostly focused on the relationship between sense of belonging and academic outcomes (Roeser et al., 1996). Very few studies have investigated students' sense of belonging as an outcome, even though research has shown that sense of belonging is an important variable in itself (Baumeister and Leary, 1995).

For negatively stereotyped ethnic minority students, sense of belonging interventions have revealed positive long-term effects on health, well-being, and performance (Walton and Cohen, 2011). Thus, belonging is an important psychological variable especially for ethnic minorities (Branscombe et al., 1999). Research has shown that a sense of belonging is also associated with the perception of fit with an academic environment (e.g., Benner and Graham, 2007; Walton and Cohen, 2007; Iyer et al., 2009) and that being together with other people from similar backgrounds can reduce depression and increase self-esteem (Schmitt and Branscombe, 2002). Having contact with other people from a similar background leads to social support and can diminish the negative effects of prejudice. This is the case because being with people from similar backgrounds can lead not only to mutual understanding but also to an increase in acceptance and sense of belonging (Miller and Major, 2000; Major and O'Brien, 2005; Fangen and Lynnebakke, 2014). In line with this reasoning, research has shown that ethnic minority students' sense of belonging to school is related to the percentage of students of the same ethnicity in academic environments. For instance, Benner and Graham (2007) found that the sense of belonging of negatively stereotyped minority students is related to change in ethnic composition at the school level during school transitions in an urban sample in the United States. Taken together, we propose that the same processes are at work for Turkish-origin students in Germany, as they are also frequently exposed to negative stereotypes (Froehlich et al., 2016a). Consequently, we hypothesize that an increase in the percentage of Turkish-origin students in the classroom will be positively related to Turkish-origin students' sense of belonging.

For ethnic majority students, research findings on the impact of ethnic composition on their sense of belonging are not consistent. Some research found that the sense of belonging of ethnic majority students transitioning to new schools was not affected by ethnic composition, because they were not reminded of being a member of an ethnic minority in the new environment (Benner and Graham, 2007). In contrast, intergroup research showed that outgroup members (e.g., migrants) can be perceived as a threat for ingroup members based on differences in values and norms (Stephan et al., 1998). Drawing on these findings, we hypothesize for German students that an increase in the proportion of Turkish-origin students in the classroom will be negatively related to their sense of belonging because they might feel threatened by the salience of Turkish-origin students in the classroom. Thus, we expect a cross-level interaction effect between the percentage of Turkish-origin students and students' ethnicity on sense of belonging.

## The present research

In the present study, we investigated ethnic classroom composition and its relationship to individual reading performance and sense of belonging to school not only for Turkish-origin but also for German students based on data from the representative National Assessment Study 2008/2009 (Böhme et al., 2010). We focused on reading performance as a dependent variable because the National Assessment Study 2008/2009 assessed only verbal competencies related to the school subject German. In contrast to previous research on ethnic composition effects in the educational context, we examined whether the ethnic composition of a specific ethnic group, namely Turkish-origin students, in the classroom is differentially related to the performance and sense of belonging of Turkish-origin and German students. We hypothesize cross-level interaction effects between the proportion of Turkish-origin students in the classroom and students' ethnicity on performance and sense of belonging. In detail, we hypothesize that the proportion of Turkish-origin students in the classroom will be negatively related to the performance of Turkish-origin students but not to the performance of German students. We further hypothesize that the proportion of Turkish-origin students in the classroom will be positively related to Turkish-origin and negatively related to German students' sense of belonging. We used multi-level regression analyses to examine the cross-level interaction effect of the increase in the percentage of Turkish-origin students on Turkish-origin and German students' reading performance and sense of belonging.

## Methods

### Data

For the data in the present study, we draw on the National Assessment Study 2008/2009 (*Ländervergleich*) in the school subject German. The National Assessment Study 2008/2009 consisted of two subsamples, which were either affiliated with the international PISA 2009 study (Programme for International Student Assessment; OECD, 2010) or exclusively recruited for the National Assessment Study 2008/2009 to enlarge the final national sample. In detail, students who participated in the international PISA study (*N* = 201 schools with two classes per school), and those students who were only recruited for the National Assessment Study 2008/2009 (*N* = 1299 schools with one class per school) comprised the final sample of the National Assessment Study 2008/2009 (*N* = 1500 schools). The sampling procedure and the data collection of the National Assessment Study 2008/2009 were conducted by the IEA-Data Processing and Research Center (IEA-DPC; for details see Böhme et al., 2010). In the National Assessment Study 2008/2009, students completed a performance test consisting of items related to reading literacy, listening comprehension, and spelling in the school subject German. Subsequently, students filled in a questionnaire which included socio-demographic data and performance- and teaching-related covariates (e.g., socioeconomic status, perception of school, sense of belonging, school grades, and perception of teacher support).

### Ethics statement

The utilization and analysis of the National Assessment Study 2008/2009 data has been approved by the Educational Quality Improvement [Institut zur Qualitätsentwicklung im Bildungswesen, IQB] and Research Data Centre [Forschungsdatenzentrum, FDZ]. Due to the representative character of the National Assessment Study 2008/2009 assessing students' competence in the school subject German, students' participation in the National Assessment Study 2008/2009 was obligatory. The parents and students were informed about the aim and procedure of the study and students' personal information was anonymized and de-identified by generating a code for each student prior to analysis. The principal investigator of the study was the IQB.

### Sample

In the present study, we focused only on students who participated exclusively in the National Assessment Study 2008/2009 because these students completed the performance test sessions and the questionnaire. Due to the representative study design of the National Assessment Study 2008/2009, the selected 15 year-old high school students should reflect the 9th grade high school student population living in Germany. After attending 4 years of elementary school, high school students in Germany attend different schools based on a school tracking system. In detail, the German education system divides high school students based on their achievement into three major school tracks: *Hauptschule* (lowest track school), *Realschule* (middle track school; in some states such as Bavaria it is also called *Mittelschule*), or *Gymnasium* (highest track school). In some federal states, an additional fourth track integrates all levels of education into one school (*Gesamtschule*), which is a comprehensive school. Only students who graduate from the highest track school can study at a university, while most students who graduate from the lowest or middle track school enter an apprenticeship (Neumann et al., 2010). Most Turkish-origin students attend either lower or middle track schools (Baier et al., 2010). In detail, 57% of Turkish-origin students attend schools of the lowest track (*Hauptschule*) in comparison to 28.3% of students without a migration background, whereas students of Turkish origin and students without migration background are equally likely to attend a middle track school (e.g., ~30% attend a *Realschule*). However, only 15.2% of Turkish-origin students in comparison to 37.4% of students without migration background attend the highest track schools (i.e., *Gymnasium*; Kristen, 2002; Baier et al., 2010). Thus, we decided to only investigate middle track high schools (*Realschule, Mittelschule*, and *Gesamtschule*), which have a similar number of Turkish-origin and German students, in order to avoid an over- or underrepresentation of Turkish-origin students as in lower and higher track schools, respectively (Baumert and Schümer, 2001). Our sample included 8702 German and 513 Turkish-origin students (48.1% female, 51.9% male; *M*_*age*_ = 15.23 years, *SD*_*age*_ = 0.65) and the mean percentage of Turkish-origin students in the classrooms was 4% with a range from 0 to 65%. Altogether, our sample represented a nested data structure, with students (*N* = 9215) nested in the classrooms (*N* = 658).

### Measures

#### Reading performance

In the present study, we focused on reading performance as a dependent variable because it is an important basic competency to acquire and implement new knowledge and thus is crucial for school success (Snow, 2002). Plausible values were generated utilizing the population parameters to estimate performance scores in the National Assessment Study 2008/2009 dataset for students' reading performance (for details, see Böhme et al., 2010). Altogether, five plausible values for the reading performance of each student were extracted (Böhme et al., 2010). We calculated the mean of all five plausible values to measure students' reading performance. Performance scores in the National Assessment Study 2008/2009 are scaled to an overall mean of 496 points across all participating federal states of Germany, with a standard deviation of 92.

#### Sense of belonging

Students' school perception was measured with eight items in the National Assessment Study 2008/2009. These items were developed for and used in the PISA study in 2000 (Kunter et al., 2002). The school perception scale consisted of items that measure to what extent students feel positively or negatively about school. As we were particularly interested in students' sense of belonging to school, based on Goodenow's (1993) definition of sense of belonging (i.e., feeling accepted, included, respected, and valued by others in school), we conducted an exploratory factor analysis with an oblique rotation on the eight items given in the dataset to measure school perception. The results of the factor analysis suggested two factors of which one reflects sense of belonging to school (see Table 1). The five items loading on this factor (item loadings ≥ 0.6) were “I feel I belong,” “I feel like an outsider” (reverse-coded), “Apparently I am popular,” “I find friends easily,” and “I feel lonely.” The second factor reflecting students' negative attitude toward school was not included in the present analyses. One item (i.e., “I often feel uncomfortable and I am out of my element”) was neither included in the first nor second factor because it loaded on both factors with comparable factor loadings. Items were rated on a 4-point-Likert scale ranging from 1 “not at all” to 4 “completely true.” The internal consistency of the 5-item sense of belonging factor was good (α = 0.80).

**Table 1:** **Loadings for the rotated factors for school perception**.

	**Factors**	
	**1**	**2**
	**Sense of belonging**	**Negative school perception**
I feel I belong.	0.77	
I feel like an outsider.	0.77	
Apparently I am popular.	0.72	
I find friends easily.	0.72	
I feel lonely.	0.71	
I often feel uncomfortable and I am out of my element.	0.64	0.51
I am often bored.		0.84
I do not want to go (to school).		0.82
Eigen value	3.38	1.38
% of variance	42.25	17.18

*Loadings < 0.4 are omitted*.

### Main predictors and covariates

Earlier research has shown that individual- and classroom-level variables are linked to students' academic performance and sense of belonging (Osterman, 2000; Walter and Stanat, 2008). We included the following predictor variables and covariates in our analyses.

#### Ethnicity

At the student level, students' migration background (i.e., Turkish or no migration background) was assessed with items asking whether the student and the parents (or known parent) were born in Turkey or Germany (Böhme et al., 2010). Moreover, we used the imputed variables for students' migration background (i.e., whether the students themselves or their parents were born abroad) which were provided in the dataset to reduce the number of missing variables. Thus, we computed a dummy variable for ethnicity from both Turkish-origin and German students' migration background variables in our analysis. Students of other ethnicities were not included in our analysis.

#### Percentage of turkish-origin students

At the classroom level, we introduced an aggregated variable describing the percentage of Turkish-origin versus non-Turkish-origin (i.e., Germans and other ethnic groups) students in the classrooms.

#### Cross-level interaction between percentage of turkish-origin students and ethnicity

We also added the cross-level interaction between ethnicity at the student level and the percentage of Turkish-origin students at the classroom level to examine the cross-level interaction effects of the Turkish-origin students' percentage on our dependent variables.

#### Student-level covariates

At the student level, various studies have demonstrated that the performance of migrant students particularly in Germany is strongly influenced by the socioeconomic status of their parents (Entorf and Minoiu, 2005). It was also shown that estimating compositional effects without controlling for individual students' prior achievement is very likely to lead to an overestimation of the effects (Goldhaber and Brewer, 1997). Further, speaking a language other than German at home is a crucial factor for migrant students' achievement (Esser, 2001). Thus, we controlled for these three factors by including as covariates parents' socioeconomic status (i.e., SES) measured by the highest International Socio-Economic Index of Occupational Status of the parents (i.e., ISEI; Ganzeboom et al., 1992), students' grade in German as a measure for previous performance, and a dummy variable for non-German languages spoken at home.

#### Classroom-level covariates

A range of studies have found that migrants' performance is also affected by SES and previous knowledge at the classroom level (e.g., Stanat et al., 2010). In addition, the proportion of other-ethnic minority students present in the classroom can influence Turkish-origin students' performance and attitude toward other ethnic groups (Thompson and Sekaquaptewa, 2002; Vervoort et al., 2011). Thus, we controlled for the proportion of other migrant students (i.e., other than Turkish-origin students) in the classrooms. Taken together, we controlled for mean SES and students' performance level by using the classroom's mean school grade in German, three middle school types (i.e., reference group: *Realschule*; *Gesamtschule*, and *Mittelschule*), and the proportion of other migrant students at the classroom level in our analyses.

### Data analysis

To test our hypothesis we followed a multilevel modeling approach to account for the nested structure of the data (i.e., students nested within classrooms). Thus, we used the Hierarchical Linear Modeling software (HLM Version 7.0; Raudenbush et al., 2011) to conduct multilevel analyses with two levels (student- and classroom-level). In so doing, we were able to differentiate effects of individual and context variables by estimating a regression equation for each school class (Snijders and Bosker, 1999). For our analyses, we conducted three successive regression models each with reading performance and sense of belonging as dependent variables. In Model 1 we examined the relationship between ethnic classroom composition (i.e., percentage of Turkish-origin students) and performance and sense of belonging, respectively, for Turkish-origin and German students. To do so, we included ethnicity (dummy-coded: Turkish-origin vs. German students) at the student level and percentage of Turkish-origin students calculated as an index (varying between 0 and 1) at the classroom level as well as the cross-level interaction between ethnicity and Turkish-origin students' percentage in this model. In Model 2, we additionally controlled for SES, grade, and non-German languages spoken at home at the student level. In Model 3, we tested whether the cross-level interaction effect remains significant when controlling for SES, grade, school type, and proportion of other migrant students at the classroom level. All dummy variables (coded 0 and 1) at both levels and the percentages of Turkish-origin and other migrant students (varying between 0 and 1) used in our models were uncentered, student-level variables were group-mean centered, and classroom-level variables were grand-mean centered. The multilevel regression model was a random-slope regression model. The equation for the model including all predictors simultaneously (Model 3) is as follows:

$$\begin{array}{l} Reading=\gamma_{00}+\gamma_{10}\cdot ethnicity_{ij}+\gamma_{20}\cdot SES_{ij}\\ performance_{ij}+\;\gamma_{30}\cdot grade_{ij}+\gamma_{40}\cdot non-German\;languages_{ij}\\ +\;\gamma_{01}\cdot percentage\;Turkish-origin\;students_{j}\\ +\;\gamma_{02}\cdot SES_{j}+\gamma_{03}\cdot grade_{j}+\gamma_{04}\cdot {\text{Gesamtschule}}_{j}\\ +\;\gamma_{05}\cdot {\text{Mittelschule}}_{j}+\gamma_{06}\cdot percentage\;of\\ other\;migrant\;students_{j}+\gamma_{11}\cdot ethnicity_{i}\\ \cdot percentage\;Turkish-origin\;students_{j}+\;u_{0j}\\ +\;u_{1j}\cdot ethnicity_{j}+u_{2j}\cdot SES_{j}+u_{3j}\cdot grade_{j}+u_{4j}\\ \cdot \;non-German\;languages_{j}+r_{ij} \end{array}$$

The indices *i* and *j* refer to students and classrooms, respectively. The same multilevel regression model was used for sense of belonging.

## Results

### Preliminary analysis

We first tested the independence of our dependent variables. The results showed that reading performance and sense of belonging were merely weakly correlated for Turkish-origin and German students, respectively (*r* = 0.09, *p* = 0.043; *r* = 0.02, *p* = 0.023). Thus, reading performance and sense of belonging can be analyzed as two independent outcomes (Cohen et al., 2013).

### Reading performance

In general, the mean reading performance differed between German (*M* = 474.61, *SD* = 65.02) and Turkish-origin students (*M* = 415.60, *SD* = 66.77) in our sample, *t*_(9213)_ = −19.95, *p* < 0.001. Both groups were below the National Assessment Study 2008/2009 overall mean of 496 points. One quarter of the variance in reading performance was due to differences between the classrooms (ICC = 0.26). In Model 1, we found a significant positive main effect of ethnicity [*b* = 48.20, *SE* = 6.29, *t*_(656)_ = 7.67, *p* < 0.001] and a marginally significant negative main effect of percentage of Turkish-origin students [*b* = −0.57.45, *SE* = 31.76, *t*_(656)_ = −1.81, *p* = 0.07], but unexpectedly no cross-level interaction effect on students' performance (*p* = 0.81; see Table 2). In Model 2, all variables reached significance (all *p*s < 0.01) except the cross-level interaction between ethnicity and percentage of Turkish-origin students (*p* = 0.44), after including students' SES, grade in German, and non-German languages spoken at home at the student level. In Model 3, similar to the results of Model 2, all variables except the cross-level interaction were significant, when we additionally controlled for SES, grade, school types, and the proportion of other migrant students at the classroom level (all *p*s < 0.01). In contrast to our hypothesis, our results did not show the predicted cross-level interaction effect of the percentage of Turkish-origin students in the classroom and ethnicity on performance (*p* = 0.51). The main effect of the classroom-level percentage of Turkish-origin students affects the performance of all students [*b* = −63.64, *SE* = 21.62, *t*_(651)_ = −2.94, *p* = 0.003]. That means a 10% increase in the proportion of Turkish-origin students in the classroom is associated with a performance decrease across all groups of 6.36 points, which indicates a rather small effect. Moreover, the main effect of the student-level ethnicity predicts performance [*b* = 26.99, *SE* = 6.19, *t*_(657)_ = 4.36, *p* < 0.001]. This indicates that higher performance is associated with German students. Model 3 explains 18.5% of the variance in reading performance among students.

**Table 2:** **Regression results for reading performance and sense of belonging**.

	**Performance**			**Sense of belonging**		
	**Model 1**	**Model 2**	**Model 3**	**Model 1**	**Model 2**	**Model 3**
**STUDENT-LEVEL**						
Intercept (γ00)	423.43^***^	443.90^***^	476.95^***^	3.23^***^	3.32^***^	3.37^***^
Ethnicity (γ10)	48.45^***^	27.62^***^	26.99^***^	0.04	−0.04	−0.04
SES (γ20)		0.24^***^	0.23^***^		< 0.01^***^	< 0.01^***^
Grade in Germans subject (γ30)		−26.69^***^	−26.64^***^		< −0.01	< −0.01
Non-German languages spoken at home (γ40)		−19.09^***^	−19.75^***^		−0.09^†^	−0.08
**CLASSROOM-LEVEL**						
Percentage of Turkish-origin students (γ01)	−57.45^†^	−83.24^**^	−63.64^**^	0.59^***^	0.55^***^	0.58^***^
Ethnicity x Percentage (γ11)	9.03	25.77	19.38	−0.62^***^	−0.56^**^	−0.56^**^
SES (γ02)			1.22^***^			< 0.01^†^
Grade in German subject (γ03)			−34.35^***^			−0.04^*^
Gesamtschule (γ04)			−29.06^***^			−0.02
Mittelschule (γ05)			−43.29^***^			−0.05^**^
Percentage of other migrant students (γ06)			−19.08^**^			−0.06
Explanatory Power (*R*^2^)	0.032	0.186	0.185	0.002	0.031	0.032

† p < 0.10;

* p < 0.05;

** p < 0.01;

*** *p < 0.01*.

### Sense of belonging

In the present study, the mean sense of belonging score differed between the two ethnic groups (Germans: *M* = 3.29, *SD* = 0.54; Turkish-origin migrants: *M* = 3.38, *SD* = 0.51), *t*_(9213)_ = 3.75, *p* > 0.001. The vast majority of variance in sense of belonging was on the individual level, with only 3% of the variance between classrooms (ICC = 0.03). In Model 1, as predicted, the main effect of percentage of Turkish-origin students [*b* = 0.59, *SE* = 0.15, *t*_(656)_ = 4.08, *p* < 0.001] and the cross-level interaction between ethnicity and percentage of Turkish-origin students reached significance [*b* = −0.62, *SE* = 0.18, *t*_(656)_ = −3.40, *p* < 0.001], but not the main effect of ethnicity (*p* = 0.30; see Table 2). In Model 2, the results of the main effect and the interaction effect of Model 1 still hold true (all *p*s < 0.01) after controlling for students' SES, grade, and non-German languages spoken at home at the student level. In Model 3, the main effect of percentage of Turkish-origin students [*b* = 0.58, *SE* = 0.12, *t*_(651)_ = 5.00, *p* < 0.001] and the cross-level interaction between ethnicity and percentage of Turkish-origin students remained significant [*b* = −0.56, *SE* = 0.16, *t*_(656)_ = −3.54, *p* < 0.001], after controlling for SES, grade, school types, and the proportion of other migrant students at the classroom level. Model 3 explains 3.2% of the variance in sense of belonging among students. The simple slope analysis (Aiken and West, 1991; Preacher et al., 2006) revealed a significant positive slope of percentage of Turkish-origin students for the sense of belonging of Turkish-origin students [*b* = 0.60, *SE* = 0.20, *t*_(651)_ = 3.03, *p* < 0.01], but not German students (*p* = 0.47). That means increasing the percentage of Turkish-origin students by 10% points increases the sense of belonging of Turkish-origin students by 0.06, which indicates a small effect. Increasing the percentage of Turkish-origin students does not affect German students' sense of belonging. To sum up, our result showed that the percentage of Turkish-origin students was positively related to Turkish-origin students' sense of belonging which is consistent with our hypothesis. Surprisingly, the Turkish-origin students' percentage was not associated with German students' sense of belonging.

## Discussion

The present study aimed at extending previous research on ethnic composition effects at the classroom level not only for Turkish-origin and German students' reading performance, but also for their sense of belonging to school. In contrast to our prediction, no cross-level interaction effect of the percent of Turkish-origin students in the classroom emerged for reading performance. However, we found a negative main effect for the percentage of Turkish-origin students in a classroom and students' ethnicity on reading performance overall, controlling for all covariates on the individual and classroom level. These results are consistent with previous studies revealing negative ethnic composition effects on Turkish-origin students' reading performance in Germany and performance differences in favor of German students (Stanat et al., 2010).

Several reasons for this finding have been discussed in the literature (Hattie, 2002; Furrer and Skinner, 2003). For instance, research found that teachers adapt their lesson plans to the perceived average classroom performance, and might be less engaged in preparing differentiated materials for low- and high-performing students (Hattie, 2002). Thus, a higher number of perceived low-performing students in the classroom might reduce the teachers' standard and thus lead to lower performance in standardized tests. Following this reasoning, it might be the case that teachers are prone to perceive classrooms with a high percentage of Turkish-origin students as low performing. This is in agreement with research showing that student teachers endorsed more negative stereotypes about the competence of Turkish-origin migrants than about Germans or Italian-origin migrants (Froehlich et al., 2016b). The student teachers attributed the underperformance of Turkish-origin students more internally (i.e., to low effort or ability) and less externally (i.e., to discrimination in the educational system) the stronger their negative stereotypes about Turkish-origin students' competence (Froehlich et al., 2016b). Therefore, it could be the case that expectations of teachers at the student and classroom level become self-fulfilling prophecies by negatively influencing teachers' performance judgments of ethnic minority students (Glock and Krolak-Schwerdt, 2013) and also influencing all students' performance expectations (Jussim and Harber, 2005). However, the main effect of the percent of Turkish-origin students in the classroom on overall classroom performance is rather small. We also found a main effect of ethnicity on performance which is in line with existing findings revealing a performance gap between German and Turkish-origin students on standardized tests (Klieme et al., 2010). Research has uncovered various reasons for this performance gap, such as Turkish-origin students' attendance of fewer cultural activities (Müller and Stanat, 2006). Further, recent studies showed that Turkish-origin students' underperformance can be caused by the activation of negative stereotypes (i.e., stereotype threat effect; Steele and Aronson, 1995) for Turkish-origin students in verbal and math domains (Martiny et al., 2015; Froehlich et al., 2016a; Mok et al., under review). A typical way to trigger stereotype threat effects is to frame a test as a diagnostic performance test (Steele and Aronson, 1995). Representative diagnostic performance test situations such as the National Assessment Study 2008/2009 might be likely to provide pressure due to the high-stakes testing situation (Sackett et al., 2004) and thus might trigger stereotype threat effects for Turkish-origin students.

As predicted, we showed a cross-level interaction on sense of belonging. The simple slope analysis showed that the percentage of Turkish-origin students in a classroom was positively related to Turkish-origin students' feelings of belonging to school. The results also showed that the percentage of Turkish-origin students was not related to German students' feelings of belonging to school. These results of sense of belonging for Turkish-origin students are important for several reasons. First, a higher sense of belonging to a school can have a positive impact on minority students' perceived fit to an academic domain (Walton and Cohen, 2007). If a higher sense of belonging among Turkish-origin students can foster a positive perceived academic fit, this might help these students to develop their full academic potential. To examine these relationships more investigations are needed. Second, research has demonstrated that sense of belonging is not only positively related to pro-social behavior, but also to well-being (Branscombe et al., 1999; Battistich et al., 2004). This means that a higher percentage of the same ethnic minority in a classroom might promote minority students' well-being. Students' sense of belonging to school has also been shown to have positive effects on self-worth later in their academic careers (Pittman and Richmond, 2007). Thus, these buffering effects on well-being might be especially important for migrant students who are confronted with discrimination based on their ethnicity (Branscombe et al., 1999) or negative stereotypes in the educational domain (Froehlich et al., 2016b). However, more research is needed to investigate these effects on well-being for Turkish-origin students. Qualitative research might help to shed light on which aspects are especially important for negatively stereotyped ethnic minority students' sense of belonging to school (Hamm and Faircloth, 2005). Further research should investigate in detail how minority students' sense of belonging is related to well-being.

Our results also highlighted that a higher percentage of Turkish-origin students in the classroom was not negatively related to German students' sense of belonging, which might at first seem surprising. One explanation can be drawn from social dominance orientation theory (Sidanius and Pratto, 2001), which argues that individuals desire their ingroup to dominate and be superior to outgroups; this dominance motivation was also shown in the academic context (Danso and Esses, 2001). In the present study, German students might perceive themselves as the higher-status group (i.e., high achieving group) in the academic domain compared to migrant students, and therefore might not feel threatened by an increased percentage of Turkish-origin students. The maintenance of dominance might explain why German students' sense of belonging to school is not associated with the percentage of Turkish-origin students. It is also likely that students who have been members of the same classroom for some time may have developed a superordinate identity as classmates (Gaertner et al., 1989) and perceive an increased homogeneity (Oakes et al., 1995). This might be particularly the case for German students because their ethnicity is less salient in the academic context and thus they might be less concerned about ethnic classroom composition (Thompson and Sekaquaptewa, 2002). Further research should examine the underlying process in detail.

## Implications

In the present study, we found a different relationship between ethnic classroom composition and performance and sense of belonging, respectively. Whereas a higher percentage of Turkish-origin students in a classroom was associated with a slightly lower reading performance for all students, it was also associated with a higher sense of belonging for Turkish-origin students. This brings us to the question of how beneficial ethnically diverse schools are for both migrant and ethnic majority students. Would students benefit from segregated schools or classrooms? In line with our finding that higher percentages of migrant students in a classroom negatively impact their performance, previous research has demonstrated negative effects of ethnically segregated environments for migrants more generally (Cutler and Glaeser, 1997; Clark and Drinkwater, 2002). Nevertheless, some studies have found positive aspects of ethnically segregated environments for migrants such as ingroup acceptance and well-being (Postmes and Branscombe, 2002). The present findings add to this ambiguous picture; we showed that an increase in the percentage of migrant students was positively associated with their sense of belonging to the school, which might positively contribute to migrant students' perception of fit in academic environments and well-being, whereas ethnic majority students' sense of belonging was not affected.

Various practical implications can be drawn. One implication is that teachers should facilitate ethnically diverse student group work in which all students feel accepted and valued by their classmates. Ethnically diverse classrooms can offer students the opportunity to get to know different cultures and reduce prejudice among both ethnic majority and minority student groups through contact during group work in the classroom (Pettigrew and Tropp, 2008). In our opinion, further research should investigate which other factors are necessary to establish a positive socio-emotional climate within classrooms and schools that also benefits all students' performance. An additional implication for teachers in ethnically diverse school settings can be drawn from the theory of culturally relevant pedagogy by Ladson-Billings (1995). The author suggested that teachers in ethnically or culturally diverse classrooms should include the cultural values and cultural knowledge of ethnically or culturally diverse students. This way of teaching might add to our understanding of what kind of support ethnic minority students need to develop a sense of belonging and well-being in the educational context (Ladson-Billings, 1995). A further implication for teachers is to rethink their teaching materials. For instance, teachers can improve their teaching materials by including different ethnic groups in their descriptions or examples. An important implication for policymakers is that more attention should be paid to increasing intercultural learning skills of future teachers within the curriculum of teacher education in Germany (Hachfeld et al., 2012). Especially given the high numbers of refugees arriving in 2015 in Germany, teachers need to be trained in intercultural learning methods to teach refugee students so they can develop their full potential. In addition, intercultural learning tasks should be implemented regularly in German school curriculum. Even though some federal states in Germany have provided initiatives to enhance intercultural learning in the school curriculum to overcome xenophobia and to support diversity (Gomolla, 2006), a national implementation of such initiatives would be a desirable advancement in the future.

## Limitations

One limitation of the present work lays in the cross-sectional nature of the data, which does not allow us to draw causal conclusions and to investigate the long-term effects of the Turkish-origin students' ethnic composition in the classroom. Investigating long-term effects is important, as most classrooms are rather stable entities over a period of several years. A longitudinal study by Stanat et al. (2010), in which the ethnic composition was operationalized by percentage of students speaking Turkish at home, found evidence that negative effects of the percentage of Turkish-origin students in the classroom on their performance was eliminated when they controlled for previous performance, SES, and school types at the classroom level. Moreover, research showed that ethnic minority students are vulnerable to classroom composition effects particularly after transitioning to a new school (Benner and Graham, 2007). Based on these findings, we believe that long-term composition effects in general and especially after students have transitioned to new schools are interesting research topics that should be addressed. Second, in our study we focused only on middle track schools in order to avoid an over- and under-representation of Turkish-origin students in lower and higher track schools (Baumert and Schümer, 2001). As our results revealed an important positive relationship between the percentage of Turkish-origin students and Turkish-origin students' sense of belonging of middle track schools, future research is needed to examine whether this positive relationship will remain stable for Turkish-origin students in lower school track classrooms.

## Conclusion

This research highlights the impact of ethnic composition on performance and sense of belonging for both migrant and non-migrant students. The present results showed that an increased percentage of Turkish-origin students in the classrooms had a small negative effect on all students' reading performance. Importantly, the findings revealed a cross-level interaction effect between students' ethnicity and the percentage of Turkish-origin students in a classroom. That means the percentage of Turkish-origin students in the classrooms was positively related to Turkish-origin students' sense of belonging but not to German students' sense of belonging. This highlights the complexity of ethnic classroom composition and diversity. As establishing multiculturalism and fostering integration of migrants is still one of the most challenging societal goals (Deaux and Verkuyten, 2014), more research should focus on the effects of ethnic composition for specific migrant groups and members of the host society. Research on ethnic classroom composition is important as it enables us to critically reflect on our educational system in order to build up an educational system in which all students—migrants and non-migrants alike—can develop their full potential and simultaneously feel a sense of belonging to their schools. Reflecting about and adapting the “mainstream” teaching strategies to the values and needs of ethnically diverse students can be a first step toward helping ethnic minority students develop a sense of belonging to school (e.g., Ladson-Billings, 1995; Hachfeld et al., 2012). In addition, sense of belonging to school can be useful for all students for coping with negative effects of stressful transitions into new environments (Iyer et al., 2009; Walton and Cohen, 2011). Thus, further research should investigate not only the conditions that hinder and foster migrant students' performance, but also psychological variables such as migrant students' sense of belonging or well-being (Branscombe et al., 1999) because these variables might also have an important impact on migrants' integration into the host society (Phinney et al., 2001).

## Author contributions

All authors listed, have made substantial, direct and intellectual contribution to the work, and approved it for publication.

## Funding

The present research was funded by the German Ministry of Education and Research (Bundesministerium für Bildung und Forschung), Grant number: 01JC1104, and also by the Graduate School of Decision Sciences of the University of Konstanz.

### Conflict of interest statement

The authors declare that the research was conducted in the absence of any commercial or financial relationships that could be construed as a potential conflict of interest.
